# The Role of Magnesium in the Secondary Phase After Traumatic Spinal Cord Injury. A Prospective Clinical Observer Study

**DOI:** 10.3390/antiox8110509

**Published:** 2019-10-24

**Authors:** André Sperl, Raban Arved Heller, Bahram Biglari, Patrick Haubruck, Julian Seelig, Lutz Schomburg, Tobias Bock, Arash Moghaddam

**Affiliations:** 1 Heidelberg Trauma Research Group, Department of Trauma and Reconstructive Surgery, Center for Orthopedics, Trauma Surgery and Spinal Cord Injury, Heidelberg University Hospital, 06221 Heidelberg, Germany; Raban.Heller@med.uni-heidelberg.de (R.A.H.); patrick.haubruck@sydney.edu.au (P.H.);; 2 Institute for Experimental Endocrinology, Charité–Universitätsmedizin Berlin, corporate member of Freie Universität Berlin, Humboldt-Universität zu Berlin, and Berlin Institute of Health, 10117 Berlin, Germany; julian.seelig@charite.de (J.S.); lutz.schomburg@charite.de (L.S.); 3 Berfsgenssenschaftliche Unfallklink Trauma Centre Ludwigshafen, Department of Paraplegiology, Head of the Department, 0621 Ludwigshafen, Germany; bahram.biglari@bgu-ludwigshafen.de; 4 Raymond Purves Bone and Joint Research Laboratories, Kolling Institute of Medical Research, Institute of Bone and Joint Research, University of Sydney, St Leonards, New South Wales, NSW 2065, Australia; 5 Aschaffenburg Trauma and Orthopedic Research Group, Center for Orthopedics, Trauma Surgery and Sports Medicine, Hospital Aschaffenburg-Alzenau, 63739 Aschaffenburg, Germany; Arash.Moghaddam-Alvandi@klinikum-ab-alz.de

**Keywords:** traumatic spinal cord injury, Magnesium, neuroprotection, neurotrauma, regeneration

## Abstract

In the secondary injury phase after traumatic spinal cord injury (TSCI), oxidative stress and neuroinflammatory responses at the site of injury constitute crucial factors controlling damage extent and may serve as potential therapeutic targets. We determined Magnesium (Mg) serum concentration dynamics in context with the potential of neurological remission in patients with TSCI as Mg is suspected to limit the production of reactive oxygen species and reduce lipid peroxidation. A total of 29 patients with acute TSCI were enrolled, and blood samples were drawn over 3 months at 11 time-points and Mg quantification was performed. Patients were divided into those with (G1, *n* = 18) or without neurological remission (G0, *n* = 11). Results show a slight drop in Mg level during the first 4 h after injury, then remained almost unchanged in G1, but increased continuously during the first 7 days after injury in G0. At day 7 Mg concentrations in G1 and G0 were significantly different (*p* = 0.039, G0 > G1). Significant differences were detected between patients in G1 that presented an AIS (ASIA Impairment Scale) conversion of 1 level versus those with more than 1 level (*p* = 0.014, G1 AIS imp. = +1 > G1 AI imp. > +1). Low and decreasing levels of Mg within the first 7 days are indicative of a high probability of neurological remission, whereas increasing levels are associated with poor neurological outcome.

## 1. Introduction

At present, there is no effective neuroprotective or neuroregenerative therapy for patients with traumatic spinal cord injury (TSCI), despite the potential devastating consequences with life-long disabilities and a permanent need of multidisciplinary treatment including surgery, medication, and long-term rehabilitation. After the initial phase of an injury, which is caused by the mechanic trauma to the spinal cord and the surrounding tissues, a secondary injury phase, including spinal shock and inflammatory responses begins [1,2,3,4,5]. According to Ditunno et al. [6] spinal shock can further be divided into four phases: (I) The first 24 hours post-injury are characterized by absent or diminished neurological reflexes. (II) Hereafter, cutaneous reflexes return between 1–3 days. (III) Then, a period of early hyperreflexia, lasting up to one month. (IV) Finally, increased spasticity and hyperreflexia of cutaneous and deep tendon reflexes characterize the final phase and can last between 1–12 months. 

In general, the final degree of impairment can only be determined after the fourth phase; whereas, clinical experience shows if no improvement of complete impairment (AIS (ASIA Impairment Scale) grade A) can be observed within 72 h after injury, chances are inferior to reach any remission [7,8], thus, making the immediate and early acute phase of the secondary injury phase after TSCI especially interesting as neuroprotective agents might be able to improve the outcome when administered during this period. Mg is the fourth most abundant cation in the human body [9]. Total and free magnesium ions (Mg^2+^) are involved in numerous cellular functions and enzymes, including ion channels, metabolic cycles, and signaling pathways [10]. For a long time, Mg concentrations were considered as stable without eliciting significant regulatory functions. Recent and compelling evidence however demonstrated significant fluxes of Mg^2+^ across the plasma membrane [10,11,12,13], affecting cell function and metabolic cycles triggered by various stimuli, such as hormones or secondary messenger-proteins. In general, Mg accumulation is mediated by Mg-channels such as TRPM6 [14] and TRPM7 [15], while outward Mg transfer is mainly operated by Mg exchangers [16,17]. In the central nervous system, although not well understood, Mg has a critical role in maintaining calcium (Ca) homeostasis and therefore is involved in neurotransmitter release, action potential conduction in nodal tissue, and transmembrane electrolyte flux. Deficiency may cause an intracellular calcium overload and disturbances in its subcellular distribution. This can lead to a stimulation of excitatory neurotransmitters such as serotonin and acetylcholine, non-competitive blockade of the *N*-methyl-d-aspartate receptor and possibly decreasing the action of the inhibitory amino acid γ-amino butyric acid [18,19]. The measurement of total Mg in serum is the most frequently used method to determine Mg levels and is already a part of the clinical routine [20]. Both in animal models of TSCI and in traumatic brain injury (TBI) in humans decreasing levels of Mg post injury could be observed and were directly linked to deterioration of secondary injury [21,22]. In combination with polyethylene glycol (Mg-PEG), Mg could enhance tissue sparing and lead to behavioral recovery in animal model [23]. The authors found remarkable clinical neuroprotection as well as vaso- and neuroprotective properties after contusion injury to the rat spinal cord. A first phase I trial (*n* = 15; NCT01750684) of an Mg-PEG combination (AC105) led by Acorda Therapeutics Inc. (Ardsley, New York, NY, USA) was concluded in February 2015 with results still pending report [24]. For this reason, we investigated the serum concentrations of Mg in adults subsequent to TSCI and tested for a possible correlation between dynamic changes in Mg levels and clinical outcomes.

## 2. Patients, Material, and Methods

### 2.1. Patient Demographics

**Figure 1:**
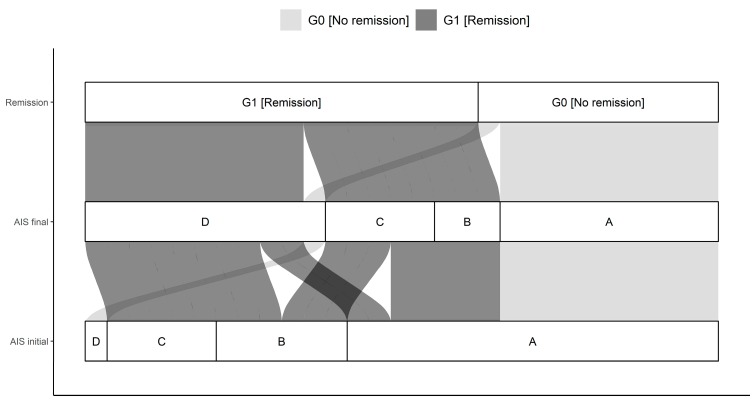
Distribution of initial AIS (association impairment scale) and final AIS in G0 and G1. The connections indicate the individual configuration of each patient with initial neurological impairment with respect to the initial and final AIS as well as the group assignment to either G0 or G1. Abb.: AIS initial = AIS level at admission, AIS final = AIS level 3 months after injury.

This study enrolled 29 patients (*n* = 21 males and *n* = 8 females) after TSCI for over 3 months. The median age of the total cohort was 43.0 (23.0, 54.0) (IQR) years and ranged between 15.0 and 75.0 years. No significant age difference between G1 (38.5 y (21.0, 56.3) and G0 44.0 y (27.0, 49.0)) could be determined. The cause of trauma was given as 19 falls (G1:11; G0:8), 8 traffic accidents (G1:2; G0:6), 1 sport accident (G1:1; G0:0), and 1 further traumatic unclassified circumstance (G1:0; G0:1). All patients were suffering from at least one fracture of the spine and were classified based on the AO-classification [25]. In particular, there were 18 A (G1:13; G0:5), 6 B (G1:2; G0:4), and 5 C (G1:3; G0:2) fractures of the spine. The neurological level of injury (NLI) is defined as the lowest neurological level where both motor and sensory function are intact. NLIs showed a distribution of 11 cervical (G1:6; G0:5), 10 thoracic (G1:5; G0:5), and 8 lumbar (G1:7; G0:1) lesions. Initial AIS grades were as following: 17 A (G1:7; G0:10), 6 B (G1:6; G0:0), 5 C (G1:5; G0:0), and 1 D (G1:0; G0:1). The final determination provided the following AIS grades: 10 A (G1:0; G0:10), 3 B (G1:3; G0:0), 5 C (G1:5; G0:0), and 11 D (G1:10; G0:1). Initial and final AIS grades differed significantly comparing G0 and G1 (*p* < 0.001) with more AIS A patients in G0 than in G1. There were no further significant differences in patients’ demographics. Further details are given in Table 1 and Table 2 and Figure 1. 

### 2.2. Material and Methods

**Figure 2:**
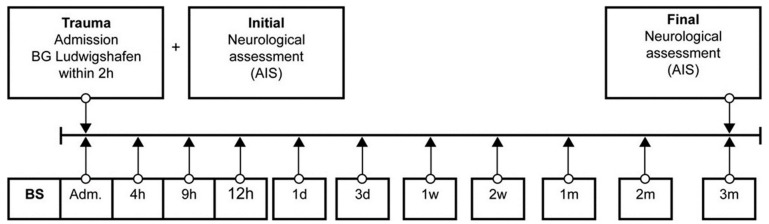
Standardized blood sample collection protocol. Abb.: BS = blood sample, Adm. = admission, AIS = American Spinal Injury Association (ASIA) Impairment Scale, h = hours, d = days, w = weeks, m = months.

Venous blood samples of 29 (21 male, 8 female) TSCI patients were collected between 2011 and 2018 in the BG Trauma Centre Ludwigshafen, Department of Paraplegiology. Exclusion criteria were non-traumatic spinal cord injury (SCI), traumatic brain injury, severe abdominal trauma, traumatic amputation of extremities, coma, or any additional significant trauma apart from SCI. Participants were not given methylprednisolone sodium succinate during study participation. During a total of 3 months, 11 blood samples were taken at specific time points after fixed protocol analog to our previous studies (Figure 2) [3,26,27,28,29,30,31,32,33,34]. 

After blood sampling and 20 min of coagulation, the blood was centrifuged at 3000 rpm, aliquoted, pseudonymized, and stored at −80 °C until analysis. Photometric determinations of Mg serum levels were conducted via the ADVIA chemistry XPT system by Siemens healthineers^®^ in an S3 laboratory of the University Heidelberg in accordance with §7 of the Genetic Engineering Act (GenTG) and Biostoffverordnung (BioStoffV). The executive laboratory staff was blinded to all patient data. To classify the neurological impairment, ASIA Impairment Scale (AIS) grades were determined by experienced examiners, according to the International Standards for Neurological Classification of SCI (spinal cord injury) (ISNCSCI; see Table 2). Initial examination (AIS initial) was performed within 72 h after admission in awake and responsive patients, final examinations (AIS final) took place at the end of the 3-months observation period. 

**Figure 3:**
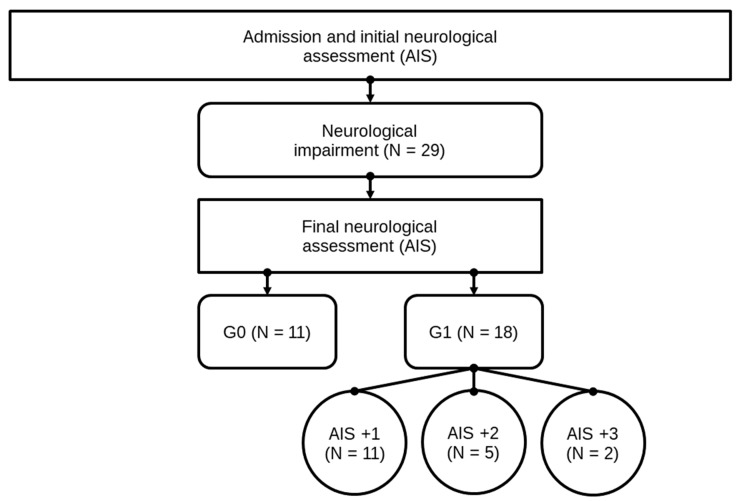
Patient collective identification scheme. Abb.: G0 / G1 = patients with (G1) or without neurological remission (G0) after 3 months, AIS +1 / AIS +2 / AIS +3 = patients with a neurological improvement of 1, 2, or 3 AIS levels within 3 months after injury.

Neurological remission was defined as an improvement of the patient’s neurological characteristics according to the AIS grades within 3 months after the trauma. Subsequently, after patients underwent the final AIS grade determination, they were divided into two study groups, consisting of 18 patients in group G1 (patients with neurological remission) and 11 patients in group G0 (patients without neurological remission). All patient data are shown in Table 1. In Figure 3, the structure of the patients collective and allocation to subgroups is presented. For a closer investigation of patients with neurological remission, subgroups of patients with an AIS conversion of 1 level (G1 AIS imp. = +1) were compared to those with an AIS level increase of more than 1 level (G1 AIS imp. > +1). A distinct pattern was recorded (Figure 5). The study was approved by the local Ethics Committee of the University of Heidelberg (S514/2011). Data collection and processing was according to good scientific practice. 

## 3. Statistical Analysis

Non-parametric test methods were assessed to investigate location shifts between (Mann-Whitney U-Test) as well as within groups at different time points (Wilcoxon Signed Rank Test). Categorical variables were evaluated using the Chi-square test. As this is an exploratory post-hoc analysis, all *p*-values are to be interpreted descriptively. No adjustment for multiple testing was adopted. All statistical calculations were performed with R version 3.6.0 [35]. Figures were created using the package ”ggplot2” [36].

## 4. Results

### 4.1. Mg-Analysis 

#### 4.1.1. Entire Patient Collective

Physiological blood serum levels of Mg vary between 0.7–1.1 mmol/L [9,18]. In this study, reference values were 0.75–1.05 mmol/L according to the manufacturer’s guidelines for the photometric determination of Mg serum levels in the central laboratory of the University of Heidelberg. Individual patient concentrations of Mg varied between 0.56 and 1.18 mmol/L.

#### 4.1.2. Comparison of Group G1 and G0 

**Figure 4:**
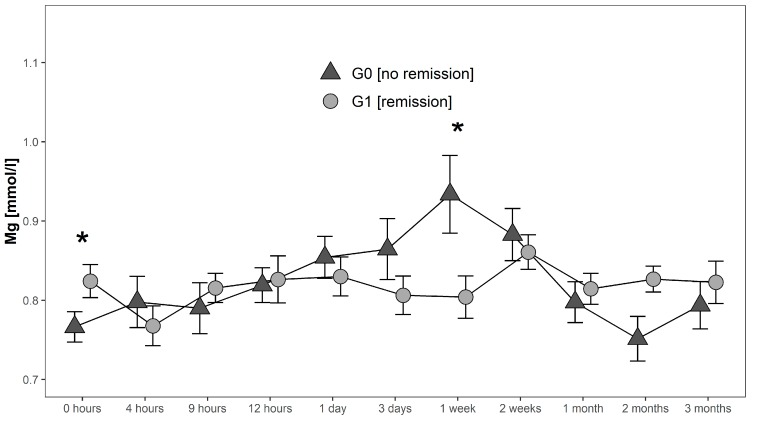
Mean Mg serum level comparison, presenting and comparing patients with (G1) and without (G0) neurological remission. Expressed as mean values ± standard error of the mean. The Mann-Whitney-U-Test assessed significant differences between both groups at each time-point (* *p* < 0.05).

Serum levels in G0 increased after initial measurements until they reached a maximum of 0.93 mmol/L 1 week after injury. Hereafter, they decreased to 0.75 mmol/L where they reached their minimum 2 months after injury. In contrast, serum Mg levels of G1 initially decreased from 0.82 mmol/L to 0.77 mmol/L in the first 4 h after injury. Then they increased again to 0.82 mmol/L at 9 h after injury to finally undulate within a range of 0.80 mmol/L 1 week after injury and 0.86 mmol/L two weeks after injury. Peak levels of Mg in G1 were detected 2 weeks after injury with a level of 0.86 mmol/L. Finally, levels decreased and stabilized between 1–3 months after trauma close to the initial levels. At admission Mg levels in G1 were significantly higher (*p* = 0.038; G1 > G0), whereas 1 week after injury serum levels of G1 were significantly lower than in G0 (*p* = 0.039) (Figure 4). 

#### 4.1.3. Comparison Within Group G1: AIS imp. = +1 and AIS imp. > +1 

**Figure 5:**
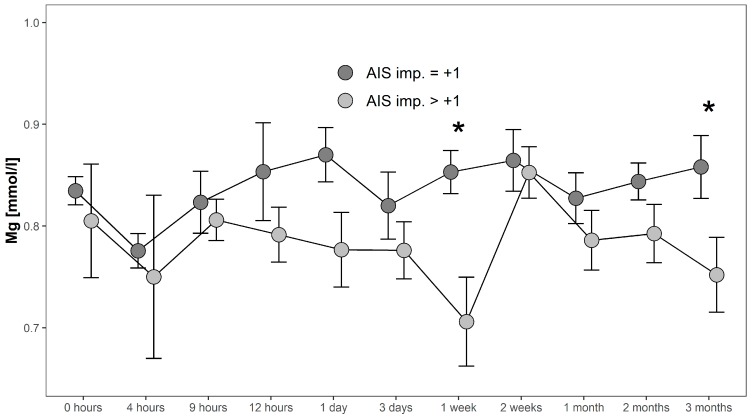
Mean Mg serum level comparison, presenting and comparing patients with neurological remission that presented an AIS conversion of 1 level (G1 AIS imp. = +1) to those with an AIS level increase of more than 1 level (G1 AIS imp. > +1) (C). Expressed as mean values ± standard error of the mean. The Mann-Whitney-U-Test assessed significant differences between both groups at each time-point (* *p* < 0.05).

**Figure 6:**
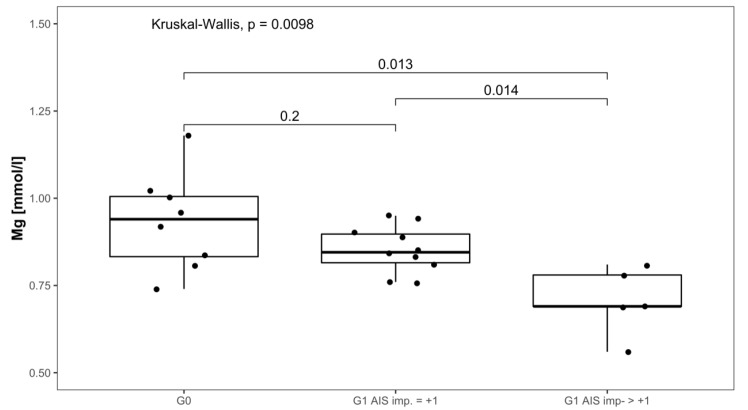
Mg serum level comparison of all patients at 1 week after injury in group G0 and patients with neurological remission that presented an AIS conversion of 1 level (G1 AIS imp. = +1) to those with an AIS level increase of more than 1 level (G1 AIS imp. > +1).

Serum Mg concentrations in patients with AIS imp. > +1 were lower throughout the 3 months recorded than in patients presenting with AIS imp. = +1. Significant differences between subgroups were found 1 week after admission (*p* = 0.014; [G1 AIS imp. = +1] > [G1 AIS imp. > +1]) and after 3 months (*p* = 0.037; [G1 AIS imp. = +1] > [G1 AIS imp. > +1]). Comparing both G1 subgroups to G0 at 1 week after injury, significant differences were detected (*p* = 0.001, Kruskal–Wallis Test, see Figure 5). The corresponding distribution can be seen in Figure 6.

## 5. Discussion

In the current study we investigated the dynamics of serum Mg concentrations in relation to the potential neurological remission in patients with TSCI. 

The results of the current study are supported by the literature. In particular, a decrease in Mg levels in the acute phase after TSCI up to 3 days post-injury has been observed in rat models. Here, Mg levels decreased in the first 24 h after injury and returned to pre-trauma levels or even above [21,37]. Because of the correlation between decreasing Mg levels and severity of the injury, Mg is suspected of playing an important role in the secondary injury phase [38,39]. In particular, Mg seems to limit the production of reactive oxygen species [40] and reduces lipid peroxidation [38,41]. In order to examine the molecular mechanisms in situations of Mg deficiency Blache et al. [40] compared standard, supplemented, and deficient Mg-diets in rats. The Mg-deficient diet-group showed a significant increase in blood pressure, plasma interleukin-6, fibrinogen, and erythrocyte lysophosphatidylcholine, and decreased plasma albumin. Further an impairment of redox status, indicated by increases in plasma thiobarbituric acid reactive substances and oxysterols and an increased blood susceptibility to in vitro free-radical-induced hemolysis was observed. The authors concluded that Mg deficiency leads to chronic impairment of redox status associated with inflammation which could significantly contribute, inter alia, to increased oxidized lipids.

In 2000, Cernak et al. found a negative correlation between Mg balance and oxidative stress in patients with TBI from severe to mild injury [22]. Similar to our study, they observed a time-dependent increase of plasma ionized Mg within the initial 7 days after injury. Conditioned by the mechanical trauma after TSCI disrupted vessels and hemorrhage at lesion side can be observed regularly. As a result, an increase in free hemoglobin (Hb) can be measured. Free Hb is known to markedly inhibit Na/K ATPase activity in CNS [42]. The decreased Na/K ATPase activity leads further to neuronal depolarization and secondary calcium influx [43]. Mg influences calcium (Ca) and glutamate levels in central nervous neurotransmission by blocking, specifically, the Ca-channel of the *N*-methyl-d-aspartate (NMDA) glutamate receptor. This inhibits Ca entry in the postsynaptic neuron which prevents excitation and thereby interrupts conduction [44]. After TBI, in situations of low Mg levels, the influx of glutamate and Ca into the postsynaptic neuron is highly elevated. Missing Mg-mediated inhibition may then lead to excitotoxicity exacerbation [38] as high levels of glutamate and Ca are strongly associated with neuronal degeneration and cell death secondary to the actual injury [45,46,47]. As Mg inhibition of NMDA receptors works in a competitive manner, the reduction of Mg levels post injury might be partially due to the binding of extracellular Mg^2+^ ions to the receptor. Although the main focus of Mg decline occurs at the lesion site, total tissue Mg falls by between 10–15% [38]. Interestingly we found significantly lower levels of Mg at 1 week after TSCI not only in G1 (remission) compared to G0 (no remission), but also in patients with an AIS level increase of more than 1 level (G1 AIS imp. > +1) compared to patients with an AIS conversion of 1 level only (G1 AIS imp. = +1). Mg levels 1 week after injury, therefore, seem to be inversely predictive of the potential for neurological remission after TSCI. Mg deficiency alone can evoke inflammatory response [48], showing an elevated release of pro-inflammatory cytokines and increased plasma levels of acute-phase proteins such as IL-6, alpha2-macroglobulin, alpha1-acid glycoprotein, and fibrinogen [49]. Furthermore, administration of MgSO_4_ reduced neutrophil invasion after SCI in a rat SCI-contusion model [50]. Given its low cost, ease of administration, the minimal risk of adverse effects in physiological concentrations and the ability to penetrate the blood-brain barrier, the neuroprotective potential of Mg is of high interest as a therapeutic approach [38]. Mg enters the central nervous system via an active transport across the blood-brain barrier (BBB) and can, therefore, be locally increased or decreased by endogenous mechanisms as required. In animal studies, a neuroprotective effect of Mg after TBI could already be detected [45,51,52,53]. In particular, the treatment has shown efficacy for neuroprotection in a dose-dependent manner [54,55,56,57]. However, neuroprotective effects in animal models could only be demonstrated when extremely high doses were used, far exceeding human tolerability. Mg in a polyethylene glycol (PEG) formulation was however found to show the same efficacy by using lower, more compatible doses [56,57]. The Mg/PEG formulation even seems beneficial compared to the treatment with methylprednisolone in terms of reduction of the lesion volume and improvement of motor outcomes [56]. In our study, we observed higher Mg serum levels between 1 day and 2 weeks after TSCI in G0 compared to G1. Mg serum levels in G1 were even significantly lower than in G0, at 1 week after injury (*p* = 0.039, Mann-Whitney-U Test). Thus, elevated Mg concentrations in the subacute phase after TSCI, seem not to be related to the neurological improvement. 

This postulation is supported by the results of animal studies. Here, Mg administration in SCI animal trials later than 8 h following injury, showed no or only small effects compared to administration within the first 8 h [56,58]. A recent Phase III double blind trial [59] in which a large cohort of 499 patients received MgSO_4_ for 5 days following TBI even showed negative outcome measures and no neuroprotective effects. In comparison, the intervention group receiving a high dose of MgSO_4_ had even a higher mortality rate than the placebo group. In contrast, Dhandapani et al. reported favorable clinical outcomes after administering MgSO_4_ within 24 h [60]. Thus, dosage and timing of administration seem to be of crucial importance to obtain a positive effect. Postnatal administration of Mg improved the neurological outcome in neonates with perinatal asphyxia with the limitation to cases of mild to moderate brain injury. In cases following severe brain injury, no effect was observed [61]. Data from the current study showed lowest Mg levels in patients with the highest remission potential (G1 AIS imp. > +1). Hence, particularly these patients might profit most from supplementary Mg. Nevertheless, at present, there is no evidence that additional Mg administration in the early phase after TSCI has a positive effect on the outcome in patients. Our study even revealed that elevated Mg levels at 1 week after injury were associated with the lack of neurological remission (Figure 4). However, the data basis is ambivalent, and additional clinical studies are needed to clarify if additional Mg administration can improve the neurological outcome. Special attention must be paid to the determination of dosage and timing.

## 6. Limitations

Despite the relevant findings, our study is not free from limitations. The main limitation was the relatively small size of the patient population. Therefore, our results must be interpreted as suggestive but before any kind of conclusions could be drawn from this, further investigations with a larger sample size are imperative. With a larger sample size for example time courses could be valued. In our study time effect on Mg is distributed nonlinear, as is the interaction between group and time. A sufficiently complex model to capture these non-linearities would require a larger sample size to fit.

## 7. Conclusions

Even though TSCI remains potentially devastating, a high percentage of patients do recover partially [8]. In our current study, we could confirm findings of decreasing Mg levels within the first 4 h after TSCI as previously demonstrated in rat models. Mg levels of patients with neurological remission were significantly lower than of the patients without remission one week after injury. Furthermore, investigation of the remission group even revealed significantly lower Mg levels in patients with AIS conversion > +1 in comparison to patients with an AIS conversion = 1 during the observation period. In synopsis, we found an inverse correlation between Mg level at 1 week after injury and the potential of neurological remission after TSCI. Considering the high complexity of processes following TSCI, it is unlikely to find a single factor which diagnostically indicates and positively affects neuroprotection and improvement of neurological outcome alone. Both for a more precise diagnosis, more reliable prediction, and better adjuvant therapy, it is likely that several factors will have to be combined into a composite parameter in order to provide the desired information and montor the clinical effects during treatment.

## Figures and Tables

**Table 1:** Demographic and clinical characteristics of subjects = study group. Abbreviations: NLI = neurological level of injury; AO = AO-classification; AIS = American Spinal Injury Association (ASIA) Impairment Scale. Age is expressed as median/mean years with their corresponding IQR and 95% CI. Neurological remission was defined as improvement in AIS within 3 months after trauma.

	All (*n* = 29)	G0 (*n* = 11)	G1 (*n* = 18)	*p*-Value
**Sex**				0.65
female	8 (28)	2 (18)	6 (33)	
male	21 (72)	9 (82)	12 (67)	
**Age**				0.62
min	15	22	15	
max	75	65	75	
median (IQR)	43 (23.00, 54.00)	44 (27.00, 49.00)	38.50 (21.00, 56.25)	
mean (95% CI)	40.69 (34.07, 47.31)	41.73 (32.72, 50.74)	40.06 (30.73, 49.38)	
**Etiology**				0.40
fall	19 (66)	8 (73)	11 (61)	
traffic	8 (28)	2 (18)	6 (33)	
other	2 (6)	1 (9)	1 (6)	
**AO**				0.23
A	18 (62)	5 (45)	13 (72)	
B	6 (21)	4 (36)	2 (11)	
C	5 (17)	2 (18)	3 (17)	
**NLI**				0.21
cervical	11 (38)	5 (45)	6 (33)	
thoracic	10 (34)	5 (45)	5 (28)	
lumbar	8 (28)	1 (9)	7 (39)	
**AIS initial**				<0.01
A	17 (59)	10 (91)	7 (39)	
B	6 (21)	0 (0)	6 (33)	
C	5 (17)	0 (0)	5 (28)	
D	1 (3)	1 (9)	0 (0)	
**AIS final**				<0.01
A	10 (34)	10 (91)	0 (0)	
B	3 (10)	0 (0)	3 (17)	
C	5 (17)	0 (0)	5 (28)	
D	11 (38)	1 (9)	10 (56)	

**Table 2:** The American Spinal Injury Association Impairment Scale (AIS). AIS grades from A–E considering the completeness of paralysis and the motor and sensory function test.

AIS Grade	Clinical State
**A**	Complete—No motor or sensory function is preserved in the sacral segments S4–S5
**B**	Incomplete—Sensory but not motor function is preserved below the NLI and includes the sacral segments S4–S5
**C**	Incomplete—Motor function is preserved below the NLI, and more than half of the key muscles below the NLI have a muscle grade less than 3
**D**	Incomplete—Motor function is preserved below the NLI, and at least half of the key muscles below the NLI have a muscle grade of 3 or more
**E**	Normal—Motor and sensory function is normal
